# Seroprevalence of Antibodies Against SARS-CoV-2 Among Health Care Workers at a Tertiary Care Hospital in Uttarakhand: A Retrospective Study

**DOI:** 10.7759/cureus.24840

**Published:** 2022-05-09

**Authors:** Amit K Saini, Prasan K Panda, Yogesh Bahurupi, Balram Omar, Akhil T, Pooja Panwar, Maneesh Sharma

**Affiliations:** 1 Department of Nursing, All India Institute of Medical Sciences, Rishikesh, Rishikesh, IND; 2 Department of Internal Medicine, All India Institute of Medical Sciences, Rishikesh, Rishikesh, IND; 3 Department of Community & Family Medicine, All India Institute of Medical Sciences, Rishikesh, Rishikesh, IND; 4 Department of Microbiology, All India Institute of Medical Sciences, Rishikesh, Rishikesh, IND

**Keywords:** sars-cov-2, covid-19, health care workers, antibodies, seroprevalence

## Abstract

Background

The number of confirmed severe acute respiratory syndrome coronavirus 2 (SARS-CoV-2) infections is vastly underestimated. In this context, seroprevalence surveys are of utmost importance to assess the proportion of the population that has already developed antibodies against the virus and might potentially be protected against subsequent infection. Health care workers (HCWs) face a greater risk of developing SARS-CoV-2. Therefore, the present retrospective study was undertaken to estimate the prevalence of antibodies against SARS-CoV-2 among healthcare workers at a tertiary care institute in Uttarakhand, India.

Material and methods

Data were gathered from hospital records of 704 healthcare workers admitted to the coronavirus disease 2019 (COVID-19) unit and attended the COVID OPD of the tertiary care institute between July 15 to Aug 14, 2020.

Result

Out of the 704 recruited participants, 14 (1.99%) were seropositive for immunoglobulin G (IgG) antibodies against SARS-CoV-2. The cumulative prevalence of SARS-CoV-2 infection (presence of antibodies or past or current positive reverse transcription-polymerase chain reaction (RT-PCR)) was 4.40%.

Conclusion

The present study shows a low prevalence of SARS-CoV-2 IgG antibodies among health care workers. In addition, posting in COVID-19-positive areas was not associated with increased seropositivity. More studies are warranted to assess IgG/IgM antibodies against SARS-CoV-2 among those HCWs who are exposed to COVID-19 patients.

## Introduction

In December 2019, a novel viral disease caused by severe acute respiratory syndrome coronavirus (SARS-CoV-2) emerged in Wuhan, China [1]. After its emergence, it rapidly spread to the whole world and posed a global threat to public health. On March 11, 2020, WHO declared coronavirus disease 2019 (COVID-19) as a pandemic, considering the severity of disease, morbidity, and mortality. The initial clinical manifestations of COVID-19 include fever, cough, headache, diarrhea, shortness of breath, nausea, vomiting, and fatigue [2]. However, the clinical syndrome may vary from mild symptoms to severe pneumonia, acute respiratory distress, and even death [3]. According to WHO's reports, globally, there were 12,964,809 cases of COVID-19 and 570,288 deaths until July 14, 2020. In the Southeast Asia region, there were 1,196,651 cases and 29,900 deaths by July 14, 2020. In India, the total numbers of cases reported were 906,752, and fatalities were 23,7272. In Uttarakhand, the total cases reported till July 15, 2020, were 3785 and deaths were 50 [4].

The COVID‑19 pandemic, caused by SARS CoV‑2, a highly transmissible virus, has placed unprecedented strain on healthcare services worldwide and surpassed other recent pandemics caused by SARS and Middle East Respiratory Syndrome (MERS) CoV [5]. SARS-CoV-2 infection is diagnosed by directly identifying viral RNA or antigens, which is considered a gold standard for active disease. Indirect identification of SARS-CoV-2 infection identifies a previous contact with the virus by detecting specific anti-SARS-CoV-2 antibodies [6]. Three main types of antibodies are produced in response to infection: immunoglobulin A (IgA), IgG, and IgM. IgM rises soonest and typically declines after infection. IgG and IgA persist and usually reflect a more extended longer-term immune response. Antibody tests look for variations in the above antibodies, either as a separate or combined antibody measurement. Antibody tests can be done in laboratory settings using enzyme-linked immunosorbent assays or chemiluminescence immunoassays (CLIA) using venous blood samples. The level of IgM antibodies begins to rise one week after the initial infection [7]. At the same time, IgG appears later than IgM (usually 14 days after infection) and can last for six months or even several years, which means that IgG serves as an indicator of the previous infection. SARS-CoV-2 can be transmitted through indirect, direct, or close contact with the infected person through oral/respiratory secretions or droplets. Airborne transmission of SARS-CoV-2 can occur by disseminating droplet nuclei (aerosols) that remain infectious when suspended in air over long distances and time [8].

Health care services are facing a significant burden because of the COVID-19 pandemic. Health care workers (HCWs) are the frontline workforce directly or indirectly involved in the clinical care of suspected and confirmed COVID-19 cases. Potential occupational exposures place HCWs at a higher risk of acquiring SARS-CoV-2 infection, which in turn may serve as an essential source of infection for their families and other community members [9]. Therefore, health care workers, such as doctors, nurses, hospital attendants, and lab technicians, are exposed to a higher risk of acquiring the disease than the general population [10-11]. To plan an adequate public health response for HCWs and anticipate the disease dynamics, the measurement of anti-SARS-CoV-2 antibodies is of utmost importance. Knowledge of the seroprevalence of SARS-CoV-2 antibodies among HCWs is essential to understanding the spread of COVID-19 among healthcare facilities and assessing public health interventions' success. Thereby, the present retrospective observational study was planned to identify COVID-19 seroprevalence among HCWs.

A preprint of this article is available on the Research Square preprint server: doi.org/10.21203/rs.3.rs-783686/v1.

## Materials and methods

The present retrospective cross-sectional study was carried out at a tertiary care hospital in Uttarakhand, between July 15 and August 14, 2020. Data were extracted to investigate study participants from e-hospital records and data available with the medical record department (MRD) by a pre-validated checklist. Hospital records of 713 health care workers admitted to the COVID-19 unit and who attended the COVID-19 OPD during this one month were extracted and analyzed. Data of various HCWs, including doctors, nurses, administrative staff, technical staff, and paramedical staff, including hospital attendants, sanitary workers, and security personnel, were included. The details of demographic characteristics, job descriptions, exposure to COVID-19, any related symptoms, prior comorbidities, and details of previous COVID-19 reverse transcription-polymerase chain reaction (RT-PCR) tests done were collected through a checklist from the questionnaire filled in by on-duty physicians during the screening OPD visit or hospital admission of HCWs. A total of nine incomplete records or missing data were excluded from the final analysis of the study. The approval for conducting the research study and data retrieving from the MRD was obtained from the Institutional Ethical Committee vide letter no AIIMS/IEC/21/18 and the competent authority of the hospital before initiating the study. RT-PCR and IgG antibody reports of all the health care workers were collected. The point prevalence of seroprevalence was measured. Data were transferred into a Microsoft Excel spreadsheet (Microsoft Corporation, Redmond, WA) and analyzed using the Statistical Package for the Social Sciences (SPSS) version 23 (IBM Corp., Armonk, NY). Frequency and percentage were used to analyze demographic and clinical variables. The chi-square and Fisher exact tests were used to determine the association of demographic and clinical variables with the serum antibody level. Also, univariate logistic regression with 95% CI that examined the prevalence of IgG and RT-PCR positivity was used.

## Results

The data of 704 health care workers were extracted from the hospital records and data available with MRD. Out of 704 participants, data of 140 participants were from the in-patient COVID-19 Unit and the data of 564 participants were from the COVID-19 OPD. There were a total of five RT-PCR reports that were inconclusive and were, therefore, excluded from analyzing prevalence and correlation with IgG antibodies. The mean age of the participants was 27.23±6.26. The majority of participants (58.9%) were male while 41.15% were female. Only 12.4% of the total participants reported that they had done duty in the COVID-positive ward in the last 28 days. Forty-one point three percent (41.3%) of participants revealed travel history to a containment zone in the past 28 days, and contact with a COVID-positive patient within the last 28 days was reported by 26% of participants. Seven point eight (7.8%), 12.6%, and 7.1% of health care workers reported fever, cough, and sore throat in the past 28 days. Only 2% of HCWs reported the presence of comorbid conditions (Table 1).

**Table 1:** Symptoms experienced by HCWs in the past 28 days (n=704) HCWs: health care workers

Symptoms	Frequency	Percentage (%)
Fever	55	7.81
Headache	3	4.26
Throat pain	89	12.64
Cough	50	7.10
Breathlessness	1	0.14
Running nose	28	3.97
Loss of taste/smell	1	0.14
Diarrhea	10	1.42
Abdominal pain	5	0.71
Bleeding tendency	3	0.42
Presence of any comorbidity	14	1.98

Of the 704 participants, 14 were seropositive for IgG antibodies against SARS-CoV-2. On RT-PCR, a total of 17 HCWs were detected positive. The cumulative prevalence of SARS-CoV-2 infection (presence of antibodies or past or current positive RT-PCR) was 4.40% among HCWs and a strong correlation was found between the RT-PCR and IgG reports (OR: 11.76; CI: 3.28-34.34) with a p-value of 0.0001 (Table 2).

**Table 2:** Association of selected variables with a seroprevalence positive test (n=704) a Independent t-test, b chi-square test, p=0.05 level of significance

Variables	Frequency/ Mean±SD		Sero-positive (n=14)	Sero-negative (n=690)	p-value
Age (Mean ±SD)^a^	27.23±6.26		28.37±6.62	26.10±5.9	0.802
Gender^b^	Male	415	5	410	0.74
	Female	289	9	280	
COVID-positive ward in last 28 days^b^	Yes	88	1	87	0.458
	No	615	13	603	
Travel history to any positive locality in the last 28 days^b^	Yes	289	7	282	0.131
	No	415	7	408	
Contact history to a positive case in the last 28 days^b^	Yes	183	1	182	0.404
	No	521	13	508	

Results depicted no significant association of the gender of the participants with an IgG-positive report (p=0.74). Similarly, visits/duties in a COVID-positive ward were not significantly associated with IgG positive reports (p=0.458). It was found that the contact of participants with a COVID-positive patient was not significantly associated with an IgG positive report (p=0.404). History of travel to a COVID-positive locality was also not significantly associated with a positive IgG report of the subjects (p=0.131) (Table 3).

**Table 3:** Prevalence of IgG and RT-PCR positivity along with their correlation (n=699) *Five RT-PCR reports were inconclusive. RT-PCR: reverse transcription-polymerase chain reaction; IgG: immunoglobulin G

IgG report		RT-PCR report				Univariable analysis (95% CI)	p-value
	Positive			Negative		11.76(3.28-34.34)	0.001
	Frequency		Percentage	Frequency	Percentage		
Positive	2		11.8 %	12	1.8%		
Negative	15		88.2%	670	98.2%		

## Discussion

HCWs are frontline personnel responsible for the clinical management of suspected or confirmed COVID-19 patients. They are at a higher risk for acquiring the disease and, if infected, pose a threat to fellow HCWs, vulnerable patients, and society. Therefore, regular screening of HCWs for SARS-CoV-2 infection is necessary to identify asymptomatic cases and exposure trends and formulate hospital policy to curb infection in the hospital setting. In the present study, 1.98% of health care workers were detected positive for IgG antibodies against SARS- COV-2, which was lower than reported studies by Hossain A et al., who showed an 8.6% seroprevalence positive rate of IgG antibodies [10]. Studies conducted by Madhusudan M et al. [6], Gupta R et al. [12], and Mishra M et al. [7] reported 16.8%, 13%, and 10.9% seropositivity to SARS-CoV-2 among HCWs. A study conducted by Anand S et al. among dialysis patients in the United States showed a seroprevalence of 8% [13]. On the contrary, a study conducted by Murhekar MV et al. on SARS-CoV-2 antibody seroprevalence in India stated that among the laboratory-confirmed COVID-19 cases, 81% of patients were having SARS-CoV-2 IgG antibodies [14], which is significantly higher in patients compared to HCWs in our study.

A comparison of serosurveillance data between HCWs and the National Centre for Disease Control (NCDC) by the Ministry of Health and Family Welfare (MoHFW) in June 2020 showed significantly higher seroprevalence in the community than in HCWs at our institute [15]. The lower seropositivity of COVID-19 infection among HCWs as compared with other places could be due to effective and vigorous training and awareness by the hospital administration, effective implementation of infection control practices in the institute, the availability and righteous use of personal protective equipment (PPE), availability of rapid diagnostic tests for early identification of cases, and prompt screening associated with contact tracing and quarantine. Posting in COVID-19-positive wards, travel history to a positive locality, and contact history with a positive case in the past 28 days was not associated with increased seropositivity in HCWs, suggesting that the isolation protocols, infection control practices, and proper use of PPE were sufficient to prevent the spread of transmission to HCWs. In the same line, a study conducted by Gupta R et al. revealed that posting in COVID-19-designated wards and the ICU was not associated with increased antibody positivity in HCWs [12]. On the contrary, in studies conducted by Rafi AM et al. [16] and Khan MS et al. [9], contact with COVID-19 patients was found to be significantly associated with seropositivity (0.001 and 0.014, respectively).

Limitations

The study was limited to health care workers working in a single tertiary care institute. Only records from July 15 to August 14, 2020, were extracted and analyzed because permission was obtained for the mentioned period only from the competent authority.

## Conclusions

We presented SARS-CoV-2 seroprevalence data in a broadly representative sample of HCWs working in a tertiary care institute in Uttarakhand and showed striking differences in seroprevalence by several HCW characteristics, with lower seroprevalence among them. Also, testing of antibodies of not only HCWs but also the general public will help take better precautionary measures if someone tests positive. It will also help the administration in making a decision and in developing new interventions and guidelines.
